# The Mediating Effect of Experiential Avoidance on the Relationship between Diabetes Distress and Self-Stigma in People with Diabetes Mellitus Type 2 in Republic of Korea

**DOI:** 10.3390/healthcare11202773

**Published:** 2023-10-20

**Authors:** Kawoun Seo

**Affiliations:** Department of Nursing, Joongbu University, Chungnam 32713, Republic of Korea; kwseo@joongbu.ac.kr

**Keywords:** diabetes mellitus, experiential avoidance, mediation analysis, stress, social stigma

## Abstract

This descriptive study aimed to explore the mediating role of experiential avoidance in the association between diabetes distress and self-stigma in Korean patients with diabetes mellitus type 2. The study included 196 participants with diabetes mellitus type 2, diagnosed by an endocrinologist. Data were collected from 20 September to 31 September 2021, using an online self-report questionnaire focusing on diabetes distress, diabetes self-stigma, and experiential avoidance. For the mediating effect analysis, a three-step hierarchical multiple analysis was performed using SPSS, and the mediating effect was verified using SPSS PROCESS Macro. The findings revealed that the average scores for diabetes distress, self-stigma, and experiential avoidance were 3.01 ± 0.66, 2.57 ± 0.82, and 3.65 ± 0.55, respectively. Positive correlations were observed among diabetes distress, self-stigma, and experiential avoidance. Specifically, experiential avoidance partially mediated the relationship between diabetes distress and self-stigma, accounting for 47.7% of the variance. These findings reveal that it is crucial to focus on countering experiential avoidance to assist patients with diabetes mellitus type 2 in overcoming the self-stigma and distress related to their condition. In addition, it is necessary to develop a gradual and tailored program aimed at reducing experiential avoidance.

## 1. Introduction

The prevalence of diabetes mellitus type 2 (T2DM) is increasing due to Western dietary habits, reduced physical activity, increased stress, and the prolongation of life expectancy. In Korea, one in seven adults over the age of 30 is a patient with T2DM, and four in ten adults over the age of 30 suffer from fasting blood sugar disorder [1]. Since T2DM is a chronic disease that requires lifelong management, patients live with the burden of constant self-care. In addition, fear of hypoglycemia, anxiety about complications occurring, mild cognitive impairment that may arise from hypoglycemia, and fear of one’s shortening life act as stressors for patients with T2DM [2]. Such stress causes patients with T2DM to experience negative emotions, such as depression, regret, anger, frustration, and loneliness, which affect their quality of life [3,4,5]. In addition, the stress experienced by patients with T2DM is a factor that worsens their condition [6]. It also interferes with glycemic control in patients with diabetes [6]. To improve the quality of life of patients with T2DM, as well as to manage their blood sugar, tackling and managing such diabetes-related stress is an essential element.

If such stresses are not managed properly, the implementation rate of self-care will be low, leading to a vicious cycle of stress from not managing T2DM [7]. Consequently, this would lead to negative thoughts not only about diabetes, but also about oneself [8]. Diabetes self-stigma reduces one’s self-esteem and self-efficacy by making the patient feel self-blame [9]. This reduced sense of self-esteem and self-efficacy reduces their quality of life, making them feel helpless for losing control over themselves, having a sense of being a social burden, and experiencing atrophy in social relationships [10]. Stress related to diabetes is unavoidable; therefore, changing the cognitive evaluation of stress is needed to prevent this stress from being transferred to diabetes self-stigma [11].

Recently, receptive behavior therapy has begun receiving attention as an intervention for reducing psychological and social stress in patients with diabetes. Acceptance and commitment therapy (ACT) is an acceptance-focused approach that enables patients to accept facts as they are, rather than judge them [12]. Where traditional cognitive behavioral therapy attempts to adaptively change a patient’s negative cognitive content, ACT focuses on cognitive functions and promotes emotion regulation strategies in response to experience avoidance [13].

Experiential avoidance refers to the tendency to avoid or change the form, frequency, and context of an unpleasant experience because it is difficult to tolerate unpleasant emotions, thoughts, memories, and bodily sensations [14]. Psychologically flexible people perceive thoughts and worries as an integral part of life, and thus tend to have lower avoidance behaviors [15,16]. In other words, even if there is a request from the same event or internal and external environment, the stress response varies according to the cognitive evaluation of an individual’s situation [17]. Specifically, individuals diagnosed with type 2 diabetes are quickly introduced to their condition and instructed in self-care practices without ample opportunity to come to terms with their diagnosis [18]. Throughout this journey, those with T2DM encounter the challenges of self-care and may initially attempt to evade these difficulties [18]. However, as they shift their focus towards problem solving, they become more proactive in effectively managing their disease [18].

Experiential avoidance is positively correlated with stress and anxiety symptoms [14,19,20]. For instance, college students’ irrational beliefs play a mediating role in influencing social anxiety [21], while experience avoidance has a mediating effect on the relationship between social anxiety and self-deprecation [21]. Studies on the relationship between diabetes distress and self-stigma have been relatively diverse. However, research on the process through which this relationship occurs is lacking.

Therefore, this study examines the relationship between diabetes distress, self-stigma, and experiential avoidance in people with T2DM. It verifies the mediating effect of experiential avoidance on the relationship between diabetes distress and self-stigma to provide fundamental data on the ACT program to reduce self-stigma in people with T2DM.

## 2. Materials and Methods

### 2.1. Study Design and Participants

In this cross-sectional study, a secondary analysis is conducted on the data gathered for the study titled “Exploring the Explanatory Model of Quality of Life: Disease Acceptance and Response Conversion in Patients with diabetes”. It explains the improvement in quality of life for people with diabetes through the acceptance of disease and a response shift to the disease. The study primarily aims to investigate the mediating role of experiential avoidance in the association between diabetes distress and self-stigma among individuals diagnosed with type 2 diabetes.

### 2.2. Participants

This study focuses on the data analyses of 200 patients with diabetes for the study titled “Quality of Life Explanatory Model through Patients with Diabetes: Disease Acceptance and Response Transformation”. The inclusion criteria for selecting participants were as follows: (1) adults aged 19 years or older, diagnosed with type 2 diabetes by a doctor; (2) individuals capable of understanding and responding to the questionnaire; and (3) participants who provided voluntary consent and completed the questionnaire. From a pool of 300 data points, a random sample of 200 was extracted and analyzed. The sample size for this study was determined using the G*power 3.1 program, specifically for a multiple regression analysis. With an effect size of 0.15 (moderate), a significance level (α) of 0.05, and a power of 0.95, a total of 178 participants was deemed necessary when utilizing diabetes distress and experience avoidance as independent variables and self-stigma as the dependent variable. Of the 200 questionnaires, 196 were included in the final analysis; the remaining 4 were excluded due to having insufficient responses.

### 2.3. Measures

#### 2.3.1. Diabetes Distress Scale

To measure diabetes distress, the Korean version of the Problem Areas in Diabetes (PAID) Scale was used [22]. This tool was developed by Polonsdy et al. [23] for stress assessments. The PAID-K comprises 20 questions, each rated on a 5-point Likert scale, ranging from 1 (strongly disagree) to 5 (strongly agree). The individual scores for all the items were summed, with higher scores indicating greater levels of diabetes-related stress. During its development, the translated PAID-K tool demonstrated a Cronbach’s α = 0.95; in this study, it exhibited a reliability of Cronbach’s α = 0.94.

#### 2.3.2. Diabetes Self-Stigma

The diabetes self-stigma measurement tool developed by Seo and Song [8] was utilized. This tool encompasses various aspects, such as relative helplessness, social withdrawal, low self-worth, and negative emotions, comprising a total of 16 items. Each item is rated on a 5-point Likert scale, ranging from 1 (strongly disagree) to 5 (strongly agree). A higher score indicates a greater level of self-stigma associated with diabetes. During its development, Seo and Song [8] reported a reliability coefficient of Cronbach’s α of 0.89 for the tool. In the present study, the tool exhibited a reliability coefficient of Cronbach’s α = 0.94.

#### 2.3.3. Experiential Avoidance

The Korean version of the Multidimensional Experience Avoidance Scale (MEAQ) was used to assess experiential avoidance. Gamez et al. [24] originally developed the MEAQ, and it was translated into Korean by Lee and You [25]. The scale comprises six sub-domains: avoidance behavior, pain aversion, delayed behavior, distraction/inhibition, repression/denial, and pain tolerance. It comprises 24 items, with each item rated on a 6-point Likert scale, ranging from 1 (strongly disagree) to 6 (strongly agree). Higher scores indicate greater levels of experiential avoidance. In the study conducted by Lee and You [25], the Cronbach’s α was 0.82, and in the present study, the tool demonstrated a reliability of Cronbach’s α = 0.84.

### 2.4. Data Collection

The data were obtained via an internet-based questionnaire administered between 20 September and 31 September 2021. This self-report survey was developed and distributed to 4300 participants associated with the research institute (PMI Co., Ltd., Seoul, Republic of Korea). From this pool, participants who acknowledged having diabetes and completed all five diabetes-related questions were chosen for further analysis. For individuals who indicated not having diabetes, the survey process was concluded automatically. Participants who completed the survey conducted for this study received compensation in accordance with the institution’s guidelines.

### 2.5. Statistical Analysis

The gathered data were processed utilizing SPSS/WIN 24.0 (IBM Corp., Armonk, NY, USA) and the SPSS PROCESS Macro (IBM Corp., Armonk, NY, USA). Initially, general characteristics and diabetes-related traits were analyzed using measures such as mean, standard deviation, frequency, and percentage. Subsequently, the correlation between diabetes distress, self-stigma, and experiential avoidance was evaluated through Pearson’s correlation coefficient. To establish the potential mediating influence of experiential avoidance on the correlation between diabetes distress and self-stigma, a three-tiered hierarchical multiple regression analysis, following Baron and Kenny’s approach [26,27], was performed. Lastly, to ascertain the significance of the mediation effect within the research model, bootstrapping was executed using the PROCESS Macro Model 4. Additionally, Cronbach’s α coefficient was computed to assess the tool’s reliability.

## 3. Results

### 3.1. General Characteristics

The participants of this study were 50.8% male, with an average age of 54.99 (±12.02) years. Regarding educational level, 65.2% had a university degree or higher, and 73.2% were married. A total of 63.6% had a job, and 41.2% engaged in social activities less than once a month. The average time since diagnosis was 7.58 (±7.76) years, and the most common type of institution where their diabetes was treated was clinics (59.6%). Those who were prescribed oral medications accounted for the majority (68.8%). The average diabetes distress was 3.01 (±0.66) and the mean self-stigma was 2.57 (±0.82). The mean of experiential avoidance was 3.65 (±0.55) (Table 1). Skewness and kurtosis were examined to assess the normality of the variables. The skewness values ranged from −0.65 to 0.85, while the kurtosis values ranged from −1.07 to 0.60. Based on these results, normality was established, since all the variables had skewness values below 3 (in absolute value) and kurtosis values below 10 (in absolute value).

### 3.2. Correlations between Diabetes Distress, Self-Stigma, and Experiential Avoidance

Diabetes distress had a significant positive correlation with both self-stigma (r = 0.43, *p* < 0.001) and experiential avoidance (r = 0.37, *p* < 0.001). This implies that, as levels of diabetes distress increased, so did levels of self-stigma and experiential avoidance. Additionally, there was a significant positive correlation between self-stigma and experiential avoidance (r = 0.26, *p* < 0.001), indicating that heightened self-stigma was associated with increased experiential avoidance (refer to Table 2).

### 3.3. Mediating Effect of Experiential Avoidance on Relationship between Diabetes Distress and Self-Stigma

**Figure 1:**
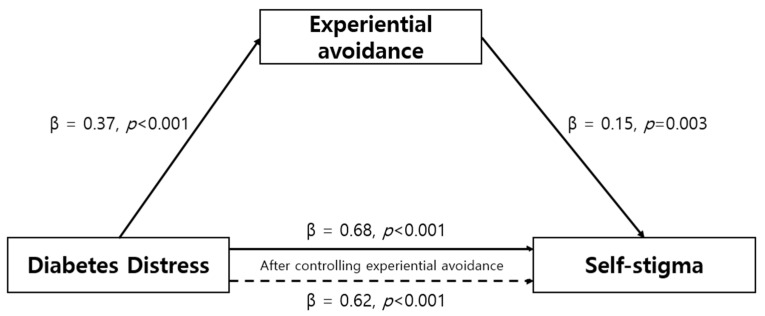
Mediating effect of experiential avoidance on the relationship between diabetes distress and self-stigma.

To evaluate the mediating effect of experiential avoidance on the relationship between diabetes distress and self-stigma among diabetes patients, a sequential three-step regression analysis was performed using the methodology outlined by Baron and Kenny [26]. Before assessing the mediating impact, the Durbin–Watson value was calculated to ensure the absence of residual autocorrelation. The result varied from 2.16 to 2.24, close to the ideal value of 2, demonstrating the absence of autocorrelation in the dependent variable. A check for multicollinearity was also performed, which revealed that all the variables had tolerance levels of 0.87 or above, within the permissible range of 1.0. Additionally, the variance inflation factor for all the variables ranged from 1.00 to 1.14, considerably below the threshold of 10, indicating no issues with multicollinearity and establishing the suitability of the regression analysis model. The outcomes of the three-step regression analysis to establish the mediating effect are detailed in Table 3. The initial step of the regression analysis confirmed a statistically significant impact of the independent variable, diabetes distress, on experiential avoidance (β = 0.38, *p* < 0.001). In the subsequent second-step regression analysis, the relationship between diabetes distress as the independent variable and self-stigma as the dependent variable was verified, demonstrating a significant association (β = 0.68, *p* < 0.001). The third step involved investigating the influence of experiential avoidance (a mediator) on the dependent variable, self-stigma. Diabetes distress and experiential avoidance were used as independent variables in this study, with self-stigma as the dependent variable. Diabetes distress (β = 0.62, *p* < 0.001) and experiential avoidance (β = 0.16, *p* = 0.004) both influenced self-stigma. When both the independent variables and the mediator are significant in the third step and the independent variable’s co-efficient is smaller than that in the second step, it suggests that the mediator has a partial mediating effect on the relationship between the independent and dependent variables [28]. In other words, the independent variable directly influences the dependent variable, while the mediator influences the dependent variable indirectly by altering the mediator’s value. The results of this study’s regression analysis indicated that both diabetes distress and experiential avoidance were significant in the third step. Furthermore, the coefficient of diabetes distress in the third step was smaller than that in the second step. A mediating effect was found, with an explanatory power of 48.6% (Table 3, Figure 1). Table 4 presents the findings from the bootstrapping analysis, highlighting the significant indirect mediating effect with an effect size of 0.06. The confidence interval further supports this significance, with the lower limit at 0.018 and the upper limit at 0.108. Importantly, the confidence interval excludes the value of 0, reinforcing the significance of the observed effect.

## 4. Discussion

This study was conducted to examine the levels of diabetes distress, self-stigma, and experiential avoidance in Korean patients with T2DM and to identify the mediating role of experiential avoidance in the relationship between diabetes distress and self-stigma.

In this study, the individuals’ diabetes distress levels averaged 3.01 points (out of 5 points). Notably, variations were found based on gender, perceived health status, type of treatment institution, and treatment methods. The diabetes distress score exceeded the average of 2.30 points in a similar study conducted by Keum et al. [29] utilizing the same assessment tool. However, although Choi and Kim [30] did not use the same tools, the results were similar to their findings, where the diabetes distress level of patients with diabetes was 6.91 points (out of 12 points). Keum et al. [29] reported that diabetes distress increases with age, but there was no difference in diabetes distress according to age in this study. Similar to this study, the subjects in Choi and Kim [30] had a shorter duration of illness compared to the subjects in Keum et al. [29]. This suggests that diabetes distress in people with diabetes is influenced more by disease-related characteristics than by age. Indeed, in this study, diabetes distress was affected by the type of hospital and treatment. Choi and Kim [30] also confirmed that the administration of oral hypoglycemic agents or insulin had a significant effect on diabetes distress [30]. Diabetes patients often experience frustration when they become aware of their condition and start taking medication, particularly insulin [31]. This realization can bring about a heightened awareness of the seriousness of the disease, leading to concerns about potential diabetes-related complications and the side effects associated with insulin use [31]. Therefore, to manage the diabetes distress of patients with T2DM, differentiated interventions are required according to the duration and characteristics of the T2DM.

The average diabetes self-stigma score was 2.57 points (out of 5 points), with variance according to perceived health status, illness duration, and treatment method. This result was similar to the findings of Seo [10], who used the same assessment tool and yielded a self-stigma score of 2.70 points. In contrast to Seo’s [10] study, which found differences in self-stigma scores depending on age, education, marital status, and employment, this study found differences in diabetes self-stigma scores based on diabetes-specific characteristics rather than general characteristics. This is thought to have been due to the differences in the participants; the participants in Seo’s [10] study were primarily female, whereas in this study, the majority of the participants were male and the age group was relatively low. In addition, although the same tool was not used, the self-stigma score was 1.82 points (out of 3 points) in a Japanese study of patients with T2DM, which was very high compared to the results of this study [32]. This can be attributed to the fact that the proportion of patients receiving insulin, with a longer average disease duration, was higher in the Japanese study than in this study [10,32]. However, an absolute comparison is not possible because the tools used to measure self-stigma were different, and self-stigma is influenced by social background. Nevertheless, it can be confirmed that diabetes self-stigma differs depending on the duration of diabetes or the type of treatment. Considering that diabetes self-stigma negatively affects the self-management of diabetes or the quality of life of patients with diabetes, continuous and focused attention was paid to the group predicted to develop self-stigma based on the results of this study. There was a positive correlation between diabetes distress and self-stigma in patients with T2DM. This means that an increase in disease-related stress in patients with T2DM has a positive effect on the increase in self-stigma. Diabetes distress refers to the emotional and behavioral burden caused by diabetes and diabetes management [33]. Such a burden generates negative emotions about diabetes and causes an unpleasant experience that generates self-stigma. However, research on diabetes self-stigma is incomplete to date. In the future, repeated follow-up studies on diabetes self-stigma, including participants with various characteristics, are needed.

The experiential avoidance score of the subjects in this study was 3.65 (out of 6), and there were differences based on whether the individuals had a spouse and a job, the degree of their social activity, and their perceived health status. This was similar to the experiential avoidance score of 3.89 in the study of Gillanders and Barker [34] in patients with diabetes. In addition, the experiential avoidance of patients with T2DM had a positive correlation with diabetes distress and self-stigma, and a mediating effect on the relationship between diabetes distress and self-stigma. In other words, if experiential avoidance appears in subjects with diabetes distress, the degree of self-stigma may increase further. In Korea, the only study on experiential avoidance in relation to disease was applied to patients with complex regional pain syndrome [35]. In this study, patients were found to inhibit or avoid activities that could pose a risk to them, because excessive perception of risk and feelings of anxiety and fear in the cognitive process led to maladaptive behavior patterns in terms of experiential avoidance [14,35]. Likewise, if patients with diabetes internalize excessive thoughts or unpleasant experiences about diabetes and feel negative emotions, it may appear as the experiential avoidance of self-care. In other words, the burden of diabetes and diabetes management experienced by patients with diabetes remains an unpleasant thought for patients with diabetes, which causes experiential avoidance in patients. Such experiential avoidance can create diabetes-related self-stigma, such as withdrawal from society or a decrease in self-efficacy or self-worth in patients with diabetes. Therefore, it is necessary to develop and apply ACT to reduce the rigidity of thinking and lead to psychological flexibility by measuring the degree of experiential avoidance in patients with diabetes. Furthermore, healthcare professionals, including nurses and medical workers, involved in the care of diabetes patients in clinical settings should assess patients’ perspectives concerning managing diabetes. Educators should also gain insights into diabetes patients’ experiences with diabetes management and work toward enhancing the understanding of these experiences. There is a clear necessity to educate nursing students about the ramifications of disease management.

Regarding limitations, the study encountered challenges in generalizing its results due to its narrow focus on a small sample of patients with diabetes. Nevertheless, the study is significant in that it investigates the correlation between diabetes distress, self-stigma, and experiential avoidance in patients with T2DM. It substantiates the idea that experiential avoidance plays a partial mediating role in the link between diabetes distress and self-stigma. These findings lay the framework for developing an ACT treatment program focused on reducing diabetes self-stigma and ultimately enhancing the well-being of individuals living with T2DM.

## 5. Conclusions

This research validated the role of experiential avoidance as a partial mediator in the relationship between diabetes distress and self-stigma among individuals diagnosed with type 2 diabetes. The results confirmed that experiential avoidance increases self-stigma in patients with diabetes when diabetes-related distress affects one’s self-stigma. Therefore, to reduce self-stigma in patients with diabetes, implementing a program to reduce experiential avoidance is necessary. Considering the findings from this research, we would like to put forth the following recommendations. First, the creation of an ACT program needs to be explored. This program would focus on mitigating experiential avoidance as an intervention to diminish self-stigma among individuals with type 2 diabetes. In the context of nursing practice, we propose the implementation of customized nursing interventions tailored to each patient’s unique circumstances. This would entail assessing the extent of self-stigma and experiential avoidance, aiding in fostering a more personalized comprehension for patients dealing with diabetes. Taking into account nursing education, we recommend that education on self-stigma and the avoidance of negative experiences be extended to individuals with diabetes, as well as nurses and nursing students. This would facilitate an enhanced understanding of the adverse psychological states stemming from diabetes.

## Figures and Tables

**Table 1:** General characteristics and independent variables of participants (N = 196).

Characteristics	Categories	M ± SD or N (%)	Min–Max
Sex	Male	127 (50.8)	
	Female	123 (49.2)	
Age (years)		54.99 ± 12.02	23.00–85.00
	≤40	31 (12.4)	
	41~64	173 (69.2)	
	≥65	46 (18.4)	
Educational level	Middle school or lower	9 (3.6)	
	High school	78 (31.2)	
	≥University or higher	163 (65.2)	
Marital status	Yes	183 (73.2)	
	No	67 (26.8)	
Having a job	Yes	159 (63.6)	
	No	91 (36.4)	
Social activities	3 or more times a week	40 (16.0)	
	Once a week	48 (19.2)	
	2~3 times a month	59 (23.6)	
	Less than once a month	10 (41.2)	
Perceived economic status	Poor	80 (32.0)	
	Moderate	113 (45.2)	
	Good	57 (22.8)	
Perceived health status	Poor	75 (30.0)	
	Moderate	115 (46.0)	
	Good	60 (24.0)	
Time since diagnosis (years)		7.58 ± 7.76	1.00–42.00
	≤3	107 (42.8)	
	4~10	70 (28.0)	
	≥11	73 (29.2)	
Type of institution where diabetes is treated	Clinic	149 (59.6)	
	General hospital	60 (24.0)	
	University hospital	39 (15.6)	
	Public health	2 (0.8)	
Type of medication	Diet therapy	43 (17.2)	
	Oral medication	172 (68.8)	
	Insulin	14 (5.6)	
	Oral medication + insulin	21 (8.4)	
Has received education on diabetes	Yes	82 (32.8)	
	No	168 (67.2)	
Diabetes distress		3.01 ± 0.66	1.25–4.50
Self-stigma		2.57 ± 0.82	1.06–4.75
Experiential avoidance		3.65 ± 0.55	1.63–5.46

M = Mean; SD = Standard deviation; Min = Minimum; and Max = Maximum.

**Table 2:** Correlations between diabetes distress, self-stigma, and experiential avoidance (N = 196).

	Diabetes Distress r (*p*)	Self-Stigma r (*p*)	Experiential Avoidance r (*p*)
Diabetes distress	1		
Self-stigma	0.43 (<0.001)	1	
Experiential avoidance	0.37 (<0.001)	0.26 (<0.001)	1

**Table 3:** Mediating effect of experiential avoidance on the relationship between diabetes distress and self-stigma (N = 196).

Step	Independent Variables	Dependent Variables	B	SE	β	t (*p*)	Adj. R^2^	F (*p*)
1	Diabetes distress	EA	0.30	0.05	0.37	6.21 (<0.001)	0.131	38.57 (<0.001)
2	Diabetes distress	Self-stigma	0.84	0.05	0.68	14.59 (<0.001)	0.460	213.02 (<0.001)
3	Diabetes distress	Self-stigma	0.77	0.06	0.621	12.69 (<0.001)	0.477	114.46 (<0.001)
	EA	Self-stigma	0.22	0.07	0.15	3.00 (<0.001)		

Adj. = Adjusted; SE = Standard error; and EA = Experiential Avoidance.

**Table 4:** Statistical significance of indirect mediation effects (N = 196).

Effect	Boot SE	95% Confidence Interval	
		Boot LLCI	BOOT ULCI
0.06	0.02	0.018	0.108

SE = standard error; LLCI = lower confidence interval; and ULCI = upper-level confidence interval.

## Data Availability

Not applicable.
